# In-Silico and In-Vitro Investigation of Key Long Non-coding RNAs Involved in 5-Fluorouracil Resistance in Colorectal Cancer Cells: Analyses Highlighting NEAT1 and MALAT1 as Contributors

**DOI:** 10.7759/cureus.66393

**Published:** 2024-08-07

**Authors:** Roxana Sahebnasagh, Zahra Azizi, Tahereh Komeili-Movahhed, Kazem Zendehdel, Mohammad Hossein Ghahremani

**Affiliations:** 1 Department of Molecular Medicine, School of Advanced Technologies in Medicine, Tehran University of Medical Sciences, Tehran, IRN; 2 Cellular and Molecular Research Center, Qom University of Medical Sciences, Qom, IRN; 3 Cancer Research Center, Cancer Institute, Tehran University of Medical Sciences, Tehran, IRN; 4 Department of Toxicology and Pharmacology, Faculty of Pharmacy, Tehran University of Medical Sciences, Tehran, IRN

**Keywords:** cerna network, 5-fu resistance, hct116, long non-coding rnas, colorectal cancer

## Abstract

Background

Acquired resistance to 5-fluorouracil (5-FU) frequently results in chemotherapy failure and disease recurrence in advanced colorectal cancer (CRC) patients. Research has demonstrated that dysregulation of long non-coding RNAs (lncRNAs) mediates the development of chemotherapy resistance in cancerous cells. The present study aims to identify key lncRNAs associated with 5-FU resistance in CRC using bioinformatic and experimental validation approaches.

Methods

The Gene Expression Omnibus (GEO) dataset GSE119481, which contains miRNA expression profiles of the parental CRC HCT116 cell line (HCT116/P) and its in-vitro established 5-FU-resistant sub-cell line (HCT116/FUR), was downloaded. Firstly, differentially expressed microRNAs (DEmiRNAs) between the parental and 5-FU resistance cells were identified. LncRNAs and mRNAs were then predicted using online databases. Gene Ontology (GO) and Kyoto Encyclopedia of Genes and Genomes (KEGG) enrichment analyses were performed to uncover relevant biological mechanisms and pathways. Networks integrating lncRNAs, miRNAs, and mRNAs interactions were constructed, and topological analyses were used to identify key lncRNAs associated with 5-FU resistance. An in-vitro model of the HCT116/FUR sub-cell line was developed by exposing the HCT116/P cell line to increasing concentrations of 5-FU. Finally, real-time quantitative PCR (RT-qPCR) was performed on total RNA extracted from the HCT116/P cell line and the HCT116/FUR sub-cell line to validate the in-silico predictions of key lncRNAs.

Results

A total of 32 DEmiRNAs were identified. Enrichment analysis demonstrated that these DEmiRNAs were mainly enriched in several cancer hallmark pathways that regulate cell growth, cell cycle, cell survival, inflammation, immune response, and apoptosis. The predictive analysis identified 237 unique lncRNAs and 123 mRNAs interacting with these DEmiRNAs. The pathway analysis indicated that most of these predicted genes were enriched in the cellular response to starvation, protein polyubiquitination, chromatin remodeling, and negative regulation of gene expression. Topological analyses of the lncRNA-miRNA-mRNA network highlighted the nuclear enriched abundant transcript 1 (NEAT1), metastasis-associated lung adenocarcinoma transcript 1 (MALAT1), and Opa interacting protein 5 antisense RNA 1 (OIP5-AS1) as central lncRNAs. Experimental analysis by RT-qPCR confirmed that the expression levels of NEAT1 and MALAT1 were significantly increased in HCT116/FUR cells compared to HCT116/P cells. However, no significant difference was observed in the OIP5-AS1 expression level between the two cells.

Conclusion

Our findings specifically highlight MALAT1 and NEAT1 as significant contributors to 5-FU resistance in CRC. These lncRNAs are promising biomarkers for diagnosing and predicting outcomes in CRC.

## Introduction

According to the National Cancer Institute, colorectal cancer (CRC) is the most common type of cancer, with an estimated 152,810 new cases and 53,010 deaths expected in 2024 [1]. Current therapeutic strategies for CRC involve surgical interventions, chemotherapy, immunotherapy, and targeted therapy. Cytotoxic chemotherapy remains the backbone of CRC management. 5-fluorouracil (5-FU) is a cytotoxic anti-cancer agent used for CRC treatment. 5-FU-based therapy was established as the standard first-line treatment in the early 2000s. It is still considered the first cycle of metastatic CRC treatment in combination with regimens such as FOLFOXIRI (folinic acid, 5-FU, oxaliplatin, and irinotecan), along with targeted therapy drugs such as bevacizumab. As a fluorinated pyrimidine analog, 5-FU functions as an antimetabolite by inhibiting thymidylate synthase, disrupting RNA and DNA synthesis, and suppressing tumor growth [2,3]. Despite its initial responses, the clinical application of 5-FU faces severe challenges due to the development of resistance, which hinders its effectiveness, contributes to tumor recurrence, and reduces the patient's survival. The mechanisms of resistance to 5-FU are complex. Studies have shown that 5-FU-resistant cells may exhibit enhanced DNA damage response to repair DNA damage caused by 5-FU. In addition, they show alterations in drug transport systems, evasion of apoptosis, dysregulation of autophagy, perturbations in the cell cycle, dysregulation of non-coding RNA expression, epigenetic alterations, epithelial-mesenchymal transition (EMT), and changes in the expression levels of thymidylate synthase and other enzymes contribute in 5-FU metabolism [3,4].

Research indicates that long non-coding RNAs (lncRNAs) are critical in mediating chemotherapy resistance in cancer. These RNA sequences, which are longer than 200 nucleotides, significantly contribute to physiological and pathological processes [5,6]. They regulate gene expression and influence multiple cellular functions, such as responses to external stimuli, DNA damage responses, cell proliferation, cell cycle regulation, apoptosis, metastasis, and cellular reprogramming. Consequently, it is expected that abnormal expression of lncRNAs is closely associated with cancer progression and chemotherapy resistance [7,8]. LncRNAs can act as competing endogenous RNAs and microRNA (miRNA) sponges, thereby regulating downstream target genes. Constructing RNA networks that include lncRNAs, miRNAs, and mRNAs provides a framework for identifying key effectors of resistance pathways and discovering potential therapeutic biomarkers [9].

To this end, our study aims to construct an integrated lncRNA-miRNA-mRNA network to identify key lncRNAs associated with 5-FU resistance in CRC. We seek to contribute to developing targeted therapeutic strategies to enhance CRC treatment.

## Materials and methods

Data acquisition

The miRNA expression profiles were retrieved from the GSE119481 dataset through the Gene Expression Omnibus (GEO) public database. This dataset, published by Gasiule S et al., includes high-throughput sequencing data obtained using the Illumina MiSeq platform (GPL15520). The study compared the miRNA expression profiles of the sensitive parental HCT116 cell line(HCT116/P) with the 5-FU-resistant HCT116 sub-cell line (HCT116/FUR). The resistant sub-cell line was developed through continuous exposure of HCT116/P cells to gradually increasing concentrations of 5-FU over nine months until stable resistance was achieved [10].

In the present study, data processing and visualization were performed using RStudio software (version 2.0.3; RStudio Team, Boston, MA) with several R packages: DESeq2 (version 1.44.0) for differential expression analysis [11], RColorBrewer (version 1.1-3) for color management, gplots (version 3.1.3.1) for various plotting functions, ggplot2 (version 3.5.1) for advanced visualizations, and org.Hs.eg.db (version 3.19.1) for gene annotation. Differentially expressed miRNAs (DEmiRNAs) were identified by comparing the HCT116/FUR sub-cell line with HCT116/P cells, using a threshold of |log2 fold change (FC)| > 0.6 and a p-value < 0.05.

Biological pathway analysis

To identify biological pathways associated with the DEmiRNAs, pathway enrichment analyses were conducted using the DIANA-miRPath tool (version 4.0; http://microrna.gr/miRPathv4/), which utilizes the Molecular Signatures Database 2022 (MSigDB) focusing on hallmark pathways [12]. The results were visualized using the online tool SRplot (http://bioinformatics.com.cn/) [13].

Predicting lncRNAs interacting with DEmiRNAs: lncRNA-miRNA interaction

To explore potential lncRNAs interacting with DE-miRNAs, two prominent online databases were used: DIANA-LncBase (release 3.0; https://diana.e-ce.uth.gr/lncbasev3/) and starBase (release 2.0; https://rnasysu.com/encori/) [14,15]. These databases are well-known for their comprehensive collections of experimentally validated lncRNA-miRNA interaction networks. Overlapping target genes were identified using a Venn diagram approach. Subsequently, the lncRNA-miRNA pairs were constructed for future analysis.

Predicting mRNAs targeted by DEmiRNAs: miRNA-mRNA interaction

The downstream target mRNAs of DE-miRNAs were predicted using multiple databases: TargetScan (release 8.0; https://www.targetscan.org/vert_80/), starBase (release 2.0; https://rnasysu.com/encori/), miRDB databases (https://mirdb.org/), and miRTarBase (release 9.0; https://miRTarBase.cuhk.edu.cn/.) [15-18]. Overlapping target genes were identified to ensure the robustness of the analysis. Subsequently, the mRNA-miRNA pairs were constructed for detailed investigation.

Enrichment analyses of predicted genes

Gene ontology (GO) analysis and Kyoto Encyclopedia of Genes and Genomes (KEGG) pathway analysis for predicted mRNAs were conducted using DAVID (version 2023q3; https://david.ncifcrf.gov/home.jsp), with criteria of count ≥ 5 and a significance threshold of p-value < 0.05 [19]. The enriched pathways and biological functions provided insights into the potential roles of predicted lncRNAs in modulating these pathways through interactions with DE-miRNAs and mRNAs. These interactions may contribute to the cancer process and the development of 5-FU resistance.

Constructing and analyzing the lncRNA-miRNA-mRNA network

Based on the predicted interactions between lncRNA-miRNA and miRNA-mRNA, a comprehensive lncRNA-miRNA-mRNA network was constructed and visualized using Cytoscape (version 3.9.1; https://cytoscape.org/). This network elucidates the potential regulatory relationships among mRNA, miRNA, and lncRNA in 5-FU resistance in CRC. Subsequently, to identify key lncRNAs within the constructed network, topological analyses were performed using the CytoNCA (version 2.1.6) plugin in Cytoscape. LncRNAs with the highest node degree (≥ 10 connections) and the highest eigenvector centrality values (indicating the influence and importance of a node's connections) were identified. These lncRNAs were considered the top three central nodes within the network, indicating their significant role in mediating 5-FU resistance in CRC.

Cell culture

The HCT116 human colorectal cancer cell line was purchased from the National Cell Bank of Iran (Pasteur Institute of Iran, Tehran, Iran). The cells were cultured in RPMI medium, supplemented with 100 U/mL penicillin, 100 µg/mL streptomycin, and 10% fetal bovine serum (all from Biowest, Nuaillé, France). Cells were cultured at 37 °C in a humidified incubator with 5% CO_2_.

Establishment of 5-FU resistance sub-cell line

The HCT116/FUR sub-cell line was established by exposing the HCT116/P cells to a gradual increase in 5-FU concentration over 10 months. Briefly, the HCT116/P cell line was cultured in a drug-free medium for 24 hours. After that, the medium was replaced with fresh medium containing 5 μM of 5-FU during the logarithmic cell growth phase. Following 72 hours of 5-FU treatment, the culture medium was replaced with a fresh, drug-free medium, and the cells were allowed to recover for two to three weeks. During this recovery period, a considerable number of drug-sensitive cells perished, while drug-resistant cells proliferated. After recovery, the culture medium was removed, the cells were trypsinized, and the remaining cells were cultured in a fresh, drug-free medium. The treatment was repeated until the cells showed stable proliferation in the presence of 5 μM of 5-FU. Subsequently, cells were exposed to increasing concentrations of 5-FU, ranging from 10 to 40 μM, and the treatment and recovery resistance induction process was repeated. Finally, cells that showed stable proliferation in the medium containing the highest concentration of 5-FU (40 μM) were considered HCT116/FUR sub-cell line. The successful induction of resistance was assessed through cytotoxicity assay and resistance index measurement, apoptosis evaluation, cell cycle analysis, and morphological observations. Cells exhibiting significant resistance compared to sensitive cells were categorized as 5-FU resistant and maintained in a drug-containing medium to sustain their resistance. Before further experiments, cells were cultured in a drug-free medium for two to three weeks.

Cytotoxicity assay

The cytotoxicity of 5-FU against the HCT116/P cell and HCT116/FUR sub-cell line was evaluated to determine the 50% inhibitory concentration (IC_50_). Briefly, cells (8× 10^3^ cells/well) were seeded in 96-well plates containing a complete drug-free medium. After 24 hours of incubation at 37 °C, the medium was replaced, and cells were treated with different concentrations of 5-FU. Following 72 hours of incubation, the medium was removed, and 20 μL of MTT solution (5 mg/mL in phosphate-buffered saline; thiazolyl blue tetrazolium bromide, Life Biolab, Germany) was added to each well. After three hours of incubation at 37 °C, formazan crystals were solubilized with 100 μL of dimethyl sulfoxide (DMSO). Finally, the optical absorbance of each well was measured at 570 nm/690 nm using an ELISA Plate Reader (Anthos, UK). The resistance index (RI) was calculated using the formula: RI = (IC_50_ of resistant sub-cell line) / (IC_50_ of the parental cell line).

Doubling-time assay

The proliferation rate of the HCT116/P cell and HCT116/FUR sub-cell line was assessed by measuring the doubling time. Briefly, cells (5×10^4^ cells/well) were seeded in 24-well plates in a complete drug-free medium. The number of cells was manually counted at 24-hour intervals for a continuous period of six days. The doubling time was calculated employing the formula: Doubling time = Duration × ln(2) / (ln(Final cell number) − ln(Initial cell number)).

Apoptosis assay

5-FU-induced apoptosis in the HCT116/P cell and HCT116/FUR sub-cell line was evaluated using flow cytometry and annexin V-FITC/propidium iodide (PI) staining. Briefly, cells (5×10^4^ cells/well) were seeded in 12-well plates in the complete drug-free medium and incubated at 37 °C for 48 hours. Then, the culture medium was removed and replaced with a fresh medium containing 40 µM of 5-FU. After 72 hours of incubation at 37 °C, the cells were harvested and washed with PBS. Subsequently, single-cell cells were re-suspended in 1X annexin binding buffer, annexin V-FITC, and PI solution (50 μg/mL), and incubated for 15 minutes at room temperature in the dark. After staining, cells were analyzed using a fluorescence-activated cell sorting Calibur flow cytometer (BD Biosciences, Madrid, Spain).

Cell cycle assay

The cell cycle distribution of the HCT116/P cell and HCT116/FUR sub-cell line exposed to 5-FU was assessed using PI staining and flow cytometry. Cells (5×10^4^ cells/well) were seeded in 12-well plates with a complete drug-free medium and incubated at 37 °C for 72 hours. After incubation, cells were then harvested and washed with cold PBS. Single cells were then resuspended in a solution containing PI, RNase (DNase-free), and PBS and left to incubate for 30 minutes in the dark at room temperature. Following staining, the distribution of cell cycle phases (G0/G1, S, and G2/M) was analyzed using a fluorescence-activated cell sorting Calibur flow cytometer.

RNA extraction and real-time qPCR (RT-qPCR)

The expression levels of the predicted lncRNAs were measured using RT-qPCR. Briefly, total RNA was extracted from the HCT116/P cell and HCT116/FUR sub-cell line using one step-RNA reagent (Bio Basic, Köln, Germany) according to the manufacturer's protocol. The concentration and quantity of RNA in all samples were determined using a spectrophotometer (US Biotek Laboratories, Shoreline, WA), with A260/280 ratios within the range of 1.8-2.0. According to the manufacturer's instructions, 1 µg of extracted RNA was reverse transcribed using the AddScript cDNA synthesis kit (Addbio, Korea) for cDNA synthesis. RT-qPCR was performed in triplicate on the StepOnePlus real-time PCR system (Thermo Fisher Scientific, Germany). Each reaction included 1 µL of template cDNA, RealQ Plus 2x Master Mix Green (Ampliqon, Odense, Denmark), and gene-specific primers. The thermal cycling conditions consisted of an initial denaturation at 95 °C for 15 minutes, followed by 40 cycles of 95 °C for 20 seconds and 60 °C for 30 seconds. Relative gene expression levels were normalized to glyceraldehyde-3-phosphate dehydrogenase (GAPDH) and calculated using the 2-ΔΔCt method. The primer pairs used for RT-qPCR reactions are detailed in Table 1. 

**Table 1:** Primers used in RT-qPCR reactions to validate the identified key lncRNAs RT-qPCR: Real-time Quantitative PCR; NEAT1: Nuclear Paraspeckle Assembly Transcript 1; MALAT1: Metastasis Associated Lung Adenocarcinoma Transcript 1; OIP5-AS1: Opa-Interacting Protein 5 Antisense RNA 1; GAPDH: Glyceraldehyde 3-Phosphate Dehydrogenase; Forward: Forward Primer; Reverse: Reverse Primer

Gene	Forward Sequences (5' →3')	Reverse Sequences (5' →3')
NEAT1	TGGCTAGCTCAGGGCTTCAG	TCTCCTTGCCAAGCTTCCTTC
MALAT1	GACGAGTTGTGCTGCTATCTT	GATTCTGTGTTATGCCTGGTTAG
OIP5-AS1	TGCGAAGATGGCGGAGTAAG	TAGTTCCTCTCCTCTGGCCG
GAPDH	GAAGGTGAAGGTCGGAGTCAAC	CAGAGTTAAAAGCAGCCCTGGT

Statistical analysis

The data were presented as mean ± standard deviation (SD). Statistical analyses were performed using GraphPad Prism 8 (GraphPad Software, LLC). A student's t-test was employed to compare the two groups. Statistical significance was defined as a p-value < 0.05.

## Results

Identification of DEmiRNAs

**Figure 1:**
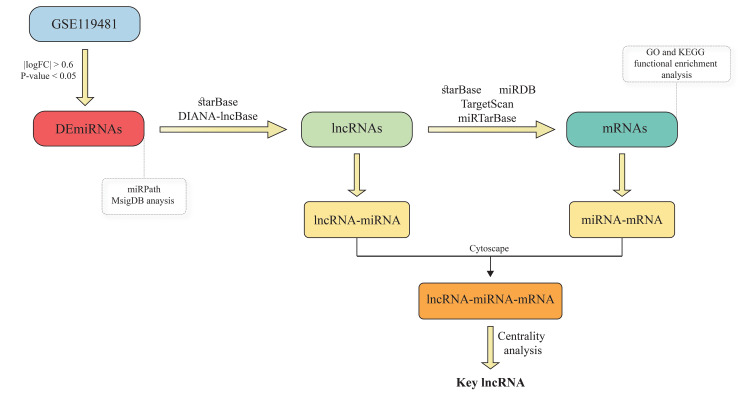
The workflow of the study process DEmiRNAs: Differentially Expressed microRNAs; lncRNA: Long Non-coding RNA; MsigDB: Molecular Signatures Database; GO: Gene Ontology; KEGG: Kyoto Encyclopedia of Genes and Genomes; logFC: Log Fold Change. Databases include miRpath, starBase, DIANA-lncBase, miRDB, TargetScan, and miRTarBase.

This study was performed as shown in the workflow in Figure 1.

Data processing of GSE119481 revealed a total of 32 DEmiRNA, including 20 upregulated and 12 downregulated, in the HCT116/FUR sub-cell line compared to the HCT116/P cell line (Table 2).

**Table 2:** List of differentially expressed microRNAs obtained from GSE119481

microRNA	log2 Fold Change	p-value	p-values adjusted
hsa-miR-224-5p	1.166782419	3.41E-14	1.20E-11
hsa-miR-589-5p	0.934383085	2.12E-08	4.97E-06
hsa-miR-218-5p	1.011987663	3.29E-08	5.78E-06
hsa-miR-27a-5p	0.93702235	6.03E-07	8.47E-05
hsa-miR-6715a-3p	4.643595446	6.85E-06	0.000687
hsa-miR-324-5p	1.267769557	1.06E-05	0.000934
hsa-miR-421	0.648026592	1.47E-05	0.001143
hsa-miR-1180-3p	0.874654306	6.09E-05	0.003291
hsa-miR-452-5p	1.141910634	6.07E-05	0.003291
hsa-miR-1258	4.126720628	0.00046	0.016148
hsa-miR-374b-3p	0.86364241	0.00055	0.017558
hsa-miR-195-5p	1.460632437	0.000703	0.021447
hsa-miR-3922-3p	3.969113156	0.000915	0.026768
hsa-miR-1303	0.975252515	0.001488	0.034812
hsa-miR-548b-5p	2.762779672	0.00176	0.037438
hsa-miR-628-5p	1.449517134	0.001712	0.037438
hsa-miR-2355-5p	2.18407414	0.001953	0.039179
hsa-miR-92b-5p	1.721664969	0.00206	0.040175
hsa-miR-324-3p	2.015381608	0.002186	0.041475
hsa-miR-30a-5p	1.087611963	0.00265	0.048957
hsa-miR-301a-5p	-1.527512674	7.45E-15	5.23E-12
hsa-miR-34a-5p	-0.711067236	6.67E-06	0.000687
hsa-miR-664a-3p	-1.41173937	4.39E-05	0.0028
hsa-miR-652-5p	-3.933398976	8.28E-05	0.004152
hsa-miR-193b-3p	-0.672519581	0.000333	0.012985
hsa-miR-197-3p	-0.769919981	0.000432	0.015963
hsa-miR-135a-5p	-3.444726388	0.00055	0.017558
hsa-miR-301b-3p	-1.253095588	0.00113	0.030509
hsa-miR-1260b	-0.871508796	0.001205	0.031337
hsa-miR-190a-5p	-1.427419981	0.001285	0.03222
hsa-miR-20a-5p	-0.625798677	0.001455	0.034812
hsa-miR-548az-5p	-2.74912666	0.001715	0.037438

**Figure 2:**
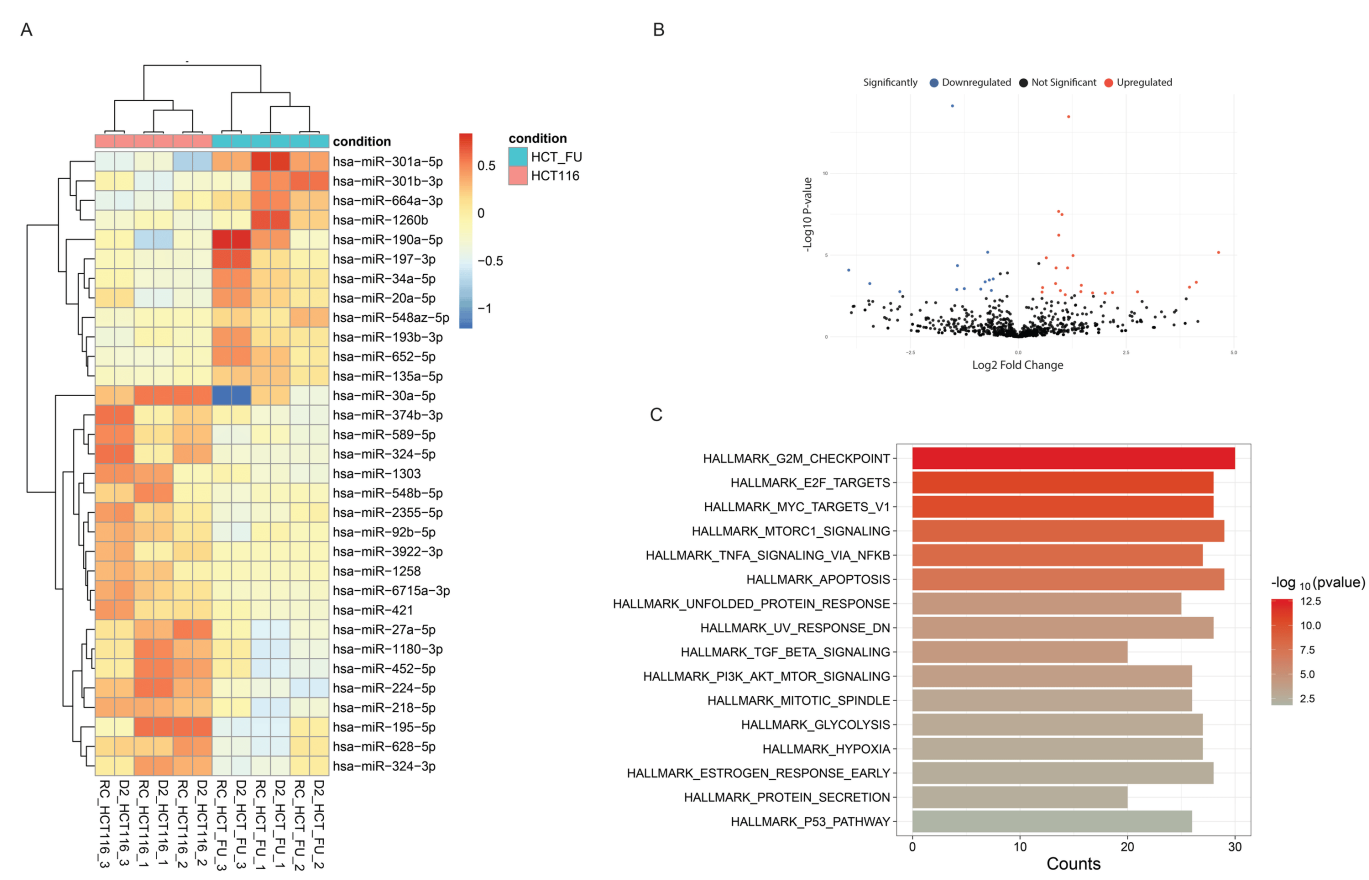
Expression patterns of DEmiRNAs in HCT116/FUR compared to the HCT116/P cell line and enrichment analysis using MsigDB (A) Heatmap: Rows represent miRNAs, and columns represent samples. Color intensity indicates relative expression levels, with blue indicating downregulation and red indicating upregulation. (B) Volcano Plot: Each point represents a miRNA plotted based on its log2 fold change (x-axis) and -log10 p-value (y-axis). Red points show significantly upregulated miRNAs, blue points show significantly downregulated miRNAs, and black points show miRNAs with no significant change. (C) MsigDB Enrichment Analysis: The horizontal axis displays various hallmark pathways involved in cellular processes. Color intensity reflects statistical significance (-log10 p-value). The vertical axis represents the number of predicted target mRNAs of miRNAs associated with each Hallmark pathway. DEmiRNAs: Differentially Expressed microRNAs; MsigDB: Molecular Signatures Database; HCT116/P: Parental HCT116 Cell Line; HCT116/FUR: 5-FU-Resistant HCT116 Sub-cell Line

Heatmap and volcano plot illustrating the expression patterns of DEmiRNAs are presented in Figures 2A-2B. The enrichment analyses of these DEmiRNAs revealed significant enrichment in multiple pathways and biological processes, including "G2/M Checkpoint", "E2F Targets", " Mammalian Target of Rapamycin Complex 1 (mTORC1) signaling", "MYC Targets," "Tumor Necrosis Factor Alpha signaling," and apoptosis (Figure 2C). These pathways indicate the potential regulatory roles of the identified DEmiRNAs in several hallmark pathways, such as cell cycle, cell growth, cell survival, inflammation, immune response, and apoptosis. The modulation of these pathways can lead to CRC progression and chemotherapy resistance.

Predicted lncRNAs and mRNAs Interacting with DEmiRNAs

**Figure 3:**
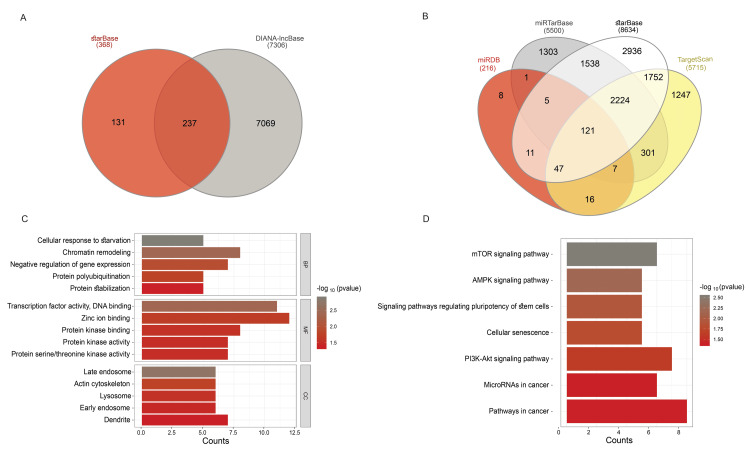
Prediction and enrichment analysis (A) Venn Diagrams: Intersection of predicted lncRNAs obtained from DIANA-LncBase and starBase databases. (B) Venn Diagrams: Intersection of predicted mRNAs from TargetScan, miRDB, starBase, and miRTarBase databases. (C) Top Five Enrichment Analyses: GO enrichment analyses are categorized into BP, CC, and MF based on fold enrichment and statistical significance (p-value < 0.05). (D) KEGG Pathway Enrichment Analyses: Enrichment of target genes in KEGG pathways. lncRNA: Long Non-coding RNA; BP: Biological Processes; CC: Cellular Components; MF: Molecular Functions; GO: Gene Ontology; KEGG: Kyoto Encyclopedia of Genes and Genomes

The intersection of predicted lncRNAs from two experimentally validated databases (DIANA-LncBase and starBase) resulted in the identification of 237 lncRNAs interacting with DEmiRNAs (Figure 3A and Table 3 in Appendices). Additionally, the intersection of predicted mRNAs from four databases (TargetScan, starBase, miRDB with a score ≥99, and miRTarBase) resulted in the identification of 121 mRNAs that are targets of DEmiRNAs (Figure 3B and Table 4 in Appendices).

KEGG pathway and GO annotation analyses were conducted to elucidate the potential biological function and signaling pathways associated with the predicted genes. The top five enriched GO terms in three classifications, including cellular components, biological processes, and molecular functions, are presented in Figure 3C. Most of the genes were enriched in "cellular response to starvation," "protein polyubiquitination," "chromatin remodeling," "negative regulation of gene expression," and "protein stabilization." In KEGG pathway analysis, significant enrichment was observed in pathways such as the "mTOR signaling," " AMP-activated protein kinase signaling," "signaling pathways regulating pluripotency of stem cells," "cellular senescence," "phosphoinositide 3-kinase-Akt (PI3K-Akt) signaling pathway," and "pathways in cancer" (p < 0.05) (Figure 3D).

Construction and topological analyses of lncRNA-miRNA-mRNA network reveals key lncRNAs associated with 5-FU resistance in CRC

**Figure 4:**
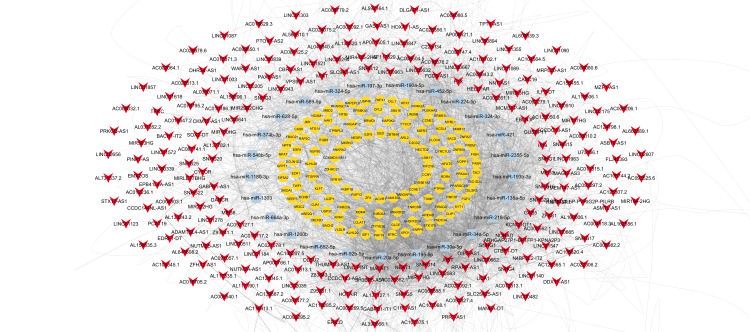
Network illustrating lncRNA-miRNA-mRNA interactions associated with 5-FU resistance in colorectal cancer Yellow circles indicate mRNAs, light blue rectangles indicate miRNAs, and red arrows indicate lncRNA. lncRNA: Long Non-coding RNA; miRNA, microRNA

The predicted interactions were used to construct the lncRNA-miRNA-mRNA regulatory network (Figure 4). Topological analyses identified nuclear enriched abundant transcript 1 (NEAT1), metastasis-associated lung adenocarcinoma transcript 1 (MALAT1), and Opa interacting protein 5 antisense RNA 1 (OIP5-AS1) as key central nodes with the highest degrees (≥ 10 connections) and significant eigenvector centrality values. These lncRNAs are considered key players in mediating 5-FU resistance in CRC.

**Figure 5:**
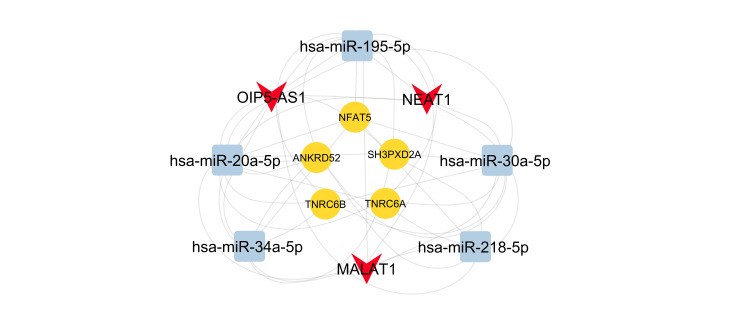
Sub-network illustrating the interactions of predicted key lncRNAs with top five miRNAs and mRNAs in the 5-FU resistance network in colorectal cancer Red arrows represent lncRNAs, yellow circles represent mRNAs, and light blue rectangles represent miRNAs. NEAT1: Nuclear Enriched Abundant Transcript 1; MALAT1: Metastasis-Associated Lung Adenocarcinoma Transcript 1; OIP5-AS1: Opa Interacting Protein 5 Antisense RNA 1; TNRC6B: Trinucleotide Repeat Containing Adaptor 6B; TNRC6A: Trinucleotide Repeat Containing Adaptor 6A; NFAT5: Nuclear Factor Of Activated T Cells 5; ANKRD52: Ankyrin Repeat Domain 52; SH3PXD2A: SH3 and PX Domains 2A

Figure 5 illustrates a sub-network that includes these three key lncRNAs, along with the top five miRNAs (hsa-miR-34a-5p, hsa-miR-195-5p, hsa-miR-20a-5p, hsa-miR-30a-5p, and hsa-miR-218-5p) and mRNAs (TNRC6B, NFAT5, TNRC6A, ANKRD52, and SH3PXD2A), all of which also exhibit high degrees (≥ 10) and significant eigenvector centrality values. This sub-network highlights potential axes associated with 5-FU resistance in CRC.

Generation and characterization of 5-FU-resistant sub-cell line

**Figure 6:**
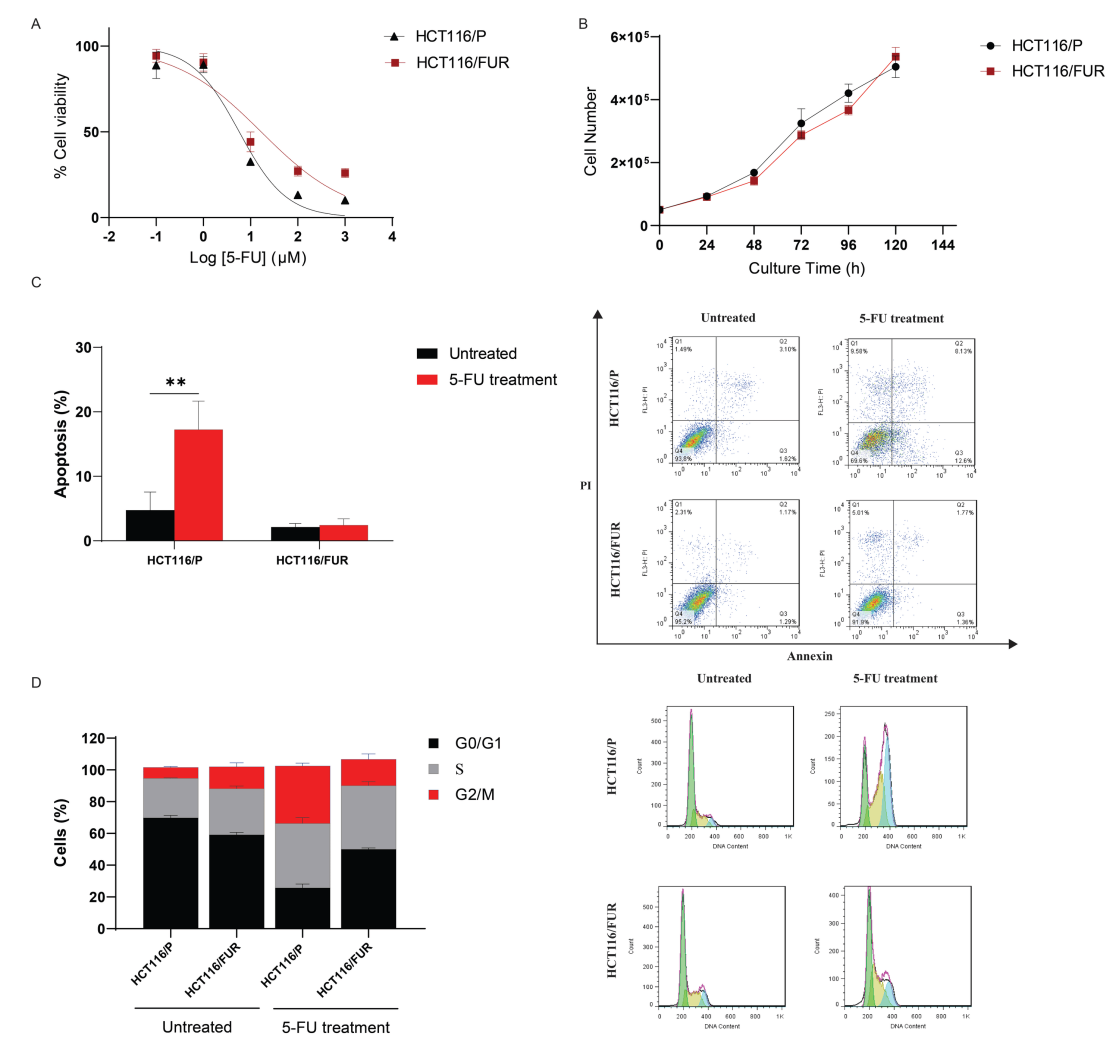
HCT116/FUR sub-cell line characterization compared with the HCT116/P cell line (A) Cell Cytotoxicity: Assessed by the MTT assay, showing that, after 72 hours of 5-FU treatment, the HCT116/FUR sub-cell line exhibited significantly greater viability compared to the parental HCT116/P cells. (B) Growth Curves: Illustrates the proliferation rate of the HCT116/FUR sub-cell line compared to the HCT116/P cell line over time. The HCT116/FUR cells exhibit a slower growth rate than the HCT116/P cells. (C) Apoptosis Histogram: Shows apoptosis induced by a 40 μM concentration of 5-FU for 72 hours, analyzed using annexin V/PI staining followed by flow cytometry. Total apoptosis is the sum of early and late apoptotic cells. Bar plots demonstrate that the HCT116/P cell line has a statistically significant apoptosis response to 5-FU compared to the HCT116/FUR sub-cell line, indicating resistance to apoptosis induction in the resistant sub-cell line. (D) Cell Cycle Distribution: Assessed by flow cytometry analysis in the HCT116/FUR sub-cell line and HCT116/P cell line following exposure to 40 μM 5-FU. The HCT116/P cells accumulate in the S and G2/M phases, whereas HCT116/FUR cells do not show as **p < 0.05.

Several assays were performed to characterize the 5-FU-resistant sub-cell line generated from the HCT116/P cell line. IC_50 _values were determined as 18.86 ± 2.16 μM for HCT116/FUR and 5.89 ± 0.19 μM for HCT116/P cells. Resistance index calculations showed a 3.2-fold more resistance to 5-FU in HCT116/FUR than in HCT116/P cells (Figure 6A). Determination of the proliferation rate revealed that the HCT116/FUR sub-cell line exhibited slower growth than the HCT116/P cell line. The doubling time in the resistant sub-cell line was extended by approximately 30 hours, representing an increase of approximately three hours compared to the HCT116/P cell line, demonstrating a doubling time of 27 hours (Figure 6B). Morphological assessment using inverted microscopy revealed a notable difference between the HCT116/FUR and HCT116/P cells. Specifically, HCT116/FUR cells appeared smaller and more rounded than the parental HCT116/P cells (data not shown). The apoptosis assay indicated that the HCT116/P cell line exhibited a significantly greater apoptosis response to 5-FU than HCT116/FUR cells when exposed to the same 5-FU concentration. However, the resistant cells resisted apoptosis induction (Figure 6C). Cell cycle distribution analysis indicated that treatment with 5-FU caused a significant decrease in the G1 phase and significant increases in both the S and G2/M phases in the HCT116/P cell line, suggesting that sensitive cells shift from the G1 phase into the S and G2/M phases. However, due to DNA damage caused by 5-FU, these cells cannot pass the G2/M checkpoint and accumulate in this phase before undergoing apoptosis. In the same condition, the HCT116/FUR sub-cell line exhibited a significant decrease in the G1 phase and an increase in the S phase. However, no significant change in the G2/M phase suggests that the resistant cells might activate mechanisms such as DNA damage repair. Despite the DNA damage, these mechanisms can potentially allow the HCT116/FUR sub-cell line to bypass the G2/M checkpoint (Figure 6D). All these observations collectively confirm the successful establishment of the HCT116/FUR sub-cell line from a parental cell line.

Association of level of NEAT1 and MALAT1 lncRNAs with 5-FU resistance in CRC cells

**Figure 7:**
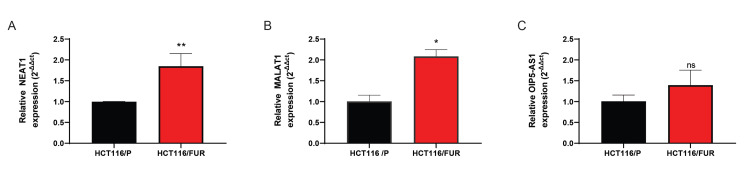
Relative expression levels of (A) NEAT1, (B) MALAT1, and (C) OIP5-AS1 in the HCT116/P cell line and HCT116/FUR sub-cell line The data are presented as means ± SD. *, **p < 0.05; ns: Not Significant; HCT116/P: Parental HCT116 Cell Line; HCT116/FUR: 5-FU-Resistant HCT116 Sub-cell Line; NEAT1: Nuclear Enriched Abundant Transcript 1; MALAT1: Metastasis-Associated Lung Adenocarcinoma Transcript 1; OIP5-AS1: Opa Interacting Protein 5 Antisense RNA 1

This study performed RT-qPCR validation explicitly for the predicted lncRNAsNEAT1, MALAT1, and OIP5-AS in the HCT116/P cell line and the HCT116/FUR sub-cell line. The results showed that the expression levels of NEAT1 and MALAT1 were significantly increased in HCT116/FUR compared to HCT116/P cells (p < 0.01 and p < 0.05, respectively). There was no significant difference in the expression level ofOIP5-AS1 between the cells (Figure 7).

## Discussion

Over the decades since the first synthesis and clinical application of 5-FU, various strategies have been developed to improve its therapeutic efficacy against CRC tumors. However, the development of resistance to 5-FU-based therapies often leads to chemotherapy failure and disease recurrence among CRC patients [3]. Reviewing published studies indicates that 5-FU resistance in CRC is a multifactorial phenomenon influenced by several mechanisms. Growing evidence demonstrates the involvement of lncRNAs in mediating 5-FU resistance in CRC. lncRNAs are critical regulatory molecules that regulate diverse cellular processes through complex lncRNA-miRNA-mRNA networks, modulating cell signaling pathways and regulating gene expression patterns. Therefore, identifying lncRNAs associated with 5-FU resistance offers potential therapeutic biomarkers to overcome drug resistance [20].

This study aimed to identify key lncRNAs involved in 5-FU resistance in CRC. Initially, miRNA expression profiles of HCT116/P and HCT116/FUR cells were obtained from the GEO dataset to identify DEmiRNAs associated with 5-FU resistance. Subsequently, bioinformatics approaches were employed to predict lncRNAs and mRNAs interacting with these DEmiRNAs. Functional enrichment analyses were performed to identify the signaling pathways that may be affected by predicted lncRNAs through their modulation of gene expression. The bioinformatics analyses also identified NEAT1, MALAT1, and OIP5-AS1 as potential key lncRNAs associated with 5-FU resistance in CRC. The subnetwork analyses showed that NEAT1, MALAT1, and OIP5-AS1 are associated with genes involved in cancer-related pathways, including the "mTOR signaling pathway," "AMPK signaling pathway", and "PI3K-Akt signaling pathway", as identified in KEGG pathway analysis. These pathways are well-known for their crucial roles in cancer progression and modulation of cancer hallmarks such as cell proliferation, apoptosis, angiogenesis, and chemoresistance [20]. These findings indicate the significant regulation of these predicted lncRNAs in cancer progression and therapeutic resistance.

In our study, experimental validation confirmed significant overexpression of NEAT1 and MALAT1 in the HCT116/FUR sub-cell line compared to the HCT116/P cell line. However, no significant difference was observed in the expression level of OIP5-AS1 between these two cell types. The lncRNA NEAT1 is a well-documented lncRNA in different types of cancer. Its elevated levels are associated with the initiation, progression, and poor prognosis of CRC. For example, Peng et al. found elevated levels of NEAT1 in both tissue and serum samples from CRC patients. They indicated that NEAT1 expression levels were directly correlated with tumor bulk, suggesting that this lncRNA facilitates cell proliferation by activating the Akt signaling pathway [21]. Our study identified NEAT1 as a central node in the lncRNA-miRNA-mRNA network associated with 5-FU resistance in CRC. Additionally, we observed significant overexpression of this lncRNA in the generated 5-FU-resistant sub-cell line compared to the parental cells. This finding aligns with other studies on various cancers, including cervical cancer, breast cancer, hepatocellular carcinoma, and notably CRC [22]. High levels of NEAT1 induce chemotherapeutic resistance by regulating several cellular processes, including DNA damage repair, cell cycle, apoptosis, autophagy, DNA damage repair, EMT, cancer stem cell characteristics, and drug metabolism and transportation [22]. As an example mechanism for NEAT1 in inducing 5-FU resistance, a study demonstrated that this lncRNA increases the expression of aldehyde dehydrogenase 1 and MYC proto-oncogene through histone acetylation and chromatin remodeling at their promoter regions. These modifications enhance these genes' transcription, promoting 5-FU resistance [23]. Considering the importance of NEAT1 in the induction of 5-FU resistance, future studies are needed to uncover its mechanisms to overcome drug resistance in CRC.

MALAT1 is one of the most notable lncRNAs found in normal tissues. It plays a complex role in several types of cancer cells where its expression levels can be upregulated or downregulated. Studies have demonstrated that MALAT1 promotes or suppresses cancer hallmarks, such as cell proliferation, metastasis, EMT, and chemoresistance [24]. Dysregulated expression of MALAT1 has been reported to be associated with chemotherapy resistance in CRC. For instance, Li et al. [15] reported high levels of MALAT1 in advanced CRC patients, correlating with poorer overall survival and reduced response to FOLFOX therapy containing leucovorin calcium, fluorouracil, and oxaliplatin [25]. In our study, we observed significant overexpression of MALAT1 in the 5-FU-resistant sub-cell line compared to its parental cells, consistent with findings in different types of cancer. A study has demonstrated that elevated MALAT1 expression is directly associated with 5-FU resistance in CRC cells. Silencing MALAT1 decreased the expression of crucial resistance proteins, including ATP-binding cassette transporters and multidrug resistance protein 1, restoring 5-FU sensitivity [26].

OIP5-AS1 has been identified as a novel and promising biomarker for multiple cancers. It functions as an oncogene and plays a critical role in tumorigenesis through the induction of cell proliferation, cell cycle, apoptosis, angiogenesis, autophagy, metastasis, EMT, invasion, and chemoresistance [27]. Previous studies have indicated that elevated levels of OIP5-AS1 induce resistance to oxaliplatin in CRC [28] and enhance resistance to cisplatin in osteosarcoma cells by activating the LPAATβ/PI3K/AKT/mTOR signaling axis [29]. However, our study did not observe significant differential expression of OIP5-AS1 between HCT116/FUR and HCT116/P cells. Nonetheless, OIP5-AS1 has been identified as a critical player in the lncRNA-miRNA-mRNA regulatory network associated with CRC 5-FU resistance. The difference in expression levels of OIP5-AS1 observed in our study may be attributed to the distinct 5-FU mechanisms of action compared to oxaliplatin, cisplatin, or other anti-cancer drugs, as well as cell-specific responses to a distinct dose of 5-FU in generating drug resistance sub-cell line, and experimental conditions. Therefore, we suggest further studies to elucidate the role of OIP5-AS1 in 5-FU resistance across different resistance levels or in various CRC cell lines.

Research has shown that NEAT1 and MALAT1 co-localize at several genomic loci, suggesting that they function independently but complement each other. They collaborate in binding and regulatory roles and interact with specific proteins [30]. Therefore, investigating the patterns of NEAT1 and MALAT1 under 5-FU resistance conditions could uncover additional mechanisms. Furthermore, examining their regulatory roles within the NEAT1-miRNA-mRNA and MALAT1-miRNA-mRNA axes identified in our constructed sub-network could further elucidate their molecular interactions under 5-FU resistance in CRC.

It is crucial to consider the limitations of the present study when interpreting the results. This research focused on a single CRC cell line and a GEO database, which may limit the generalizability of the findings. Additional CRC cell lines and GEO databases based on high-throughput data are recommended to investigate more key lncRNAs associated with 5-FU resistance. The study also relied solely on an in-vitro model. More in-vitro models, in-vivo models, and clinical samples are needed to understand and confirm the potential of predicted key lncRNAs as biomarkers in CRC treatment.

## Conclusions

In summary, we predicted the potential involvement of key lncRNAs NEAT1, MALAT1, and OIP5-AS1 in 5-FU resistance in CRC. Experimental validation confirmed significant overexpression of NEAT1 and MALAT1 in the successfully generated HCT116/FUR sub-cell line compared to the HCT116/P cells. These lncRNAs are extensively involved in critical malignancy behaviors, such as proliferation, invasion, and resistance in CRC. The presented results introduce NEAT1 and MALAT1 as valuable biomarkers for diagnosing 5-FU resistance in CRC.

## Appendices

**Table 3:** Predicted long non-coding RNAs interacting with DEmiRNAs

lncRNA	lncRNA	lncRNA	lncRNA
ZFHX2-AS1	MIR100HG	ERICD	AC107375.1
ZFAS1	MIAT	EPB41L4A-AS1	AC107068.1
Z95331.1	MEG8	EMX2OS	AC106820.4
Z93241.1	MEG3	EDRF1-DT	AC106744.2
Z83843.1	MCM3AP-AS1	EBLN3P	AC105345.1
XIST	MANEA-DT	DLGAP1-AS1	AC104564.5
WARS2-AS1	MALAT1	DLEU2	AC103702.1
VPS9D1-AS1	MAGI2-AS3	DHRS4-AS1	AC099850.1
UCA1	LINC-PINT	DDX11-AS1	AC093827.4
U73166.1	LINC02656	DANCR	AC093525.6
TPT1-AS1	LINC02607	CYTOR	AC093297.2
TMEM147-AS1	LINC02593	CTBP1-DT	AC093010.2
THUMPD3-AS1	LINC02175	CKMT2-AS1	AC092747.4
TERC	LINC02035	CCDC18-AS1	AC092295.2
STX17-AS1	LINC01963	CCDC183-AS1	AC092279.1
STAG3L5P-PVRIG2P-PILRB	LINC01857	CCDC144NL-AS1	AC092127.1
SOX2-OT	LINC01618	CBR3-AS1	AC091057.1
SNHG7	LINC01572	CASC19	AC087741.1
SNHG5	LINC01547	C22orf34	AC087477.2
SNHG4	LINC01521	C1RL-AS1	AC084125.2
SNHG3	LINC01355	BACH1-IT2	AC078795.2
SNHG29	LINC01303	ASMTL-AS1	AC068888.1
SNHG25	LINC01278	ASB16-AS1	AC026362.1
SNHG22	LINC01184	ARHGAP27P1-BPTFP1-KPNA2P3	AC026356.1
SNHG20	LINC01140	AP006621.1	AC024075.2
SNHG17	LINC01123	AP001505.1	AC022613.1
SNHG16	LINC01090	AP000766.1	AC022306.2
SNHG15	LINC01089	AL731537.2	AC021092.1
SNHG14	LINC01087	AL662889.1	AC021078.1
SNHG12	LINC01003	AL645608.2	AC020978.6
SNHG1	LINC00963	AL603839.3	AC018647.2
SLC9A3-AS1	LINC00943	AL592164.1	AC016876.2
SLC25A25-AS1	LINC00910	AL583810.1	AC016717.2
SH3BP5-AS1	LINC00894	AL390066.1	AC016705.2
RPARP-AS1	LINC00847	AL162586.1	AC016629.3
RNF216P1	LINC00839	AL160006.1	AC015871.3
PTOV1-AS2	LINC00667	AL158835.3	AC015813.1
PRR7-AS1	LINC00665	AL158206.1	AC012313.1
PRKCQ-AS1	LINC00662	AL138820.1	AC012236.1
PINK1-AS	LINC00641	AL137782.1	AC011815.1
PCAT19	LINC00632	AL137127.1	AC010186.3
PAX8-AS1	LINC00511	AL133243.2	AC009779.2
OIP5-AS1	LINC00482	AL118506.1	AC009269.5
NUTM2B-AS1	LINC00339	AL117335.1	AC009032.1
NUTM2A-AS1	LINC00294	AL117190.1	AC008982.2
NORAD	LINC00205	AL049840.4	AC006504.5
NNT-AS1	KCNQ1OT1	AL035071.1	AC006206.1
NEAT1	ILF3-DT	AL031282.2	AC005562.1
N4BP2L2-IT2	HOXA11-AS	AF117829.1	AC005540.1
MZF1-AS1	HOTAIR	ADAMTSL4-AS1	AC005261.1
MRPL20-AS1	HELLPAR	AC156455.1	AC004943.2
MIRLET7BHG	H19	AC145207.5	AC004918.3
MIR9-3HG	GUSBP11	AC139887.2	AC004803.1
MIR503HG	GAS6-AS1	AC135050.6	AC004080.6
MIR4435-2HG	GAS5	AC126365.1	AC004080.5
MIR29B2CHG	GABPB1-IT1	AC125807.2	AC003092.1
MIR222HG	GABPB1-AS1	AC124798.1	AC002064.1
MIR194-2HG	FTX	AC124045.1	
MIR17HG	FLJ42393	AC116913.1	
MIR137HG	FGD5-AS1	AC110285.2	

**Table 4:** Predicted mRNAs interacting with DEmiRNAs

mRNA	mRNA	mRNA	mRNA
MIER3	EIF5A2	SMIM13	BRMS1L
LCLAT1	VOPP1	NR3C2	ITGB8
XPO1	RNF38	BACH2	VLDLR
SNX16	KIF21B	FASN	TXNIP
PIP4K2A	HECTD2	FGF2	KCNB1
NT5E	CLIP1	TNRC6B	CLOCK
FNDC3A	GJA1	PTPN4	RUFY2
EED	SKIDA1	PHF19	ARHGAP12
SH3PXD2A	ESR1	UBE2Q1	STK17B
BRWD1	ACVR1	LSM11	RAB22A
CCNE2	TSC1	FBXO21	USP46
NCAM1	RPS6KA5	CCNE1	C2CD2
RFX7	MDM4	ATG14	EZH1
KLHL28	KLF7	LUZP1	ITPRIPL2
TNRC6A	IGF1	ARIH1	REEP3
PRDM1	RAP2C	BTRC	RRAGD
USP37	ACSL4	ARL2	PLEKHA3
ZFX	MYBL1	CASK	CFL2
DLL1	DCUN1D3	NUP50	AAK1
FKBP1B	NFAT5	CYB561A3	SACS
SYT1	TWF1	ZBTB46	ZNF827
E2F5	KLHL20	ANKRD52	MAP3K2
PPARGC1A	PPARGC1B	ZFYVE26	GPR161
MBD2	MKRN3	ENPP5	JMY
FAM135A	ANKRA2	FYCO1	GLCE
FIGN	LIN28B	DYNC1LI2	RABGAP1L
ZDHHC21	RORA	ZNFX1	COMMD3-BMI1
NEGR1	PTGFRN	NAPEPLD	NPTN
PRKAA1	CELSR3	NPAT	GFPT1
CCNY	ARID4B	TBC1D20	ZNF800
MEX3D			
